# Educational intervention to improve the knowledge of hand hygiene in pediatric residents and nurses

**DOI:** 10.12669/pjms.35.3.388

**Published:** 2019

**Authors:** Muhammad Faheem Afzal, Muhammad Haroon Hamid, Azra Parveen, Asif Hanif

**Affiliations:** 1 *Dr. Muhammad Faheem Afzal, FCPS, MHPE., Department of Pediatrics, King Edward Medical University, Lahore, Pakistan*; 2 *Dr. Muhammad Haroon Hamid, FCPS., Department of Pediatrics, King Edward Medical University, Lahore, Pakistan*; 3 *Dr. Azra Parveen, FCPS. Department of Infectious Diseases, Shaukat Khanum Memorial Cancer Hospital & Research Centre, Lahore, Pakistan*; 4 *Asif Hanif, PhD. University Institute of Public Health Faculty of Allied Health Sciences, The University of Lahore, Lahore – Pakistan*

**Keywords:** Educational intervention, Hand hygiene, Knowledge, Nurses, Pediatric residents

## Abstract

**Objectives:**

To assess the improvement in the knowledge of hand hygiene in Pediatric residents and nurses after theoretical and hands-on educational intervention

**Methods:**

This study was a questionnaire-based cross-sectional survey carried out in the department of Pediatrics, King Edward Medical University/ Mayo hospital Lahore in two weeks period. Total 41 Pediatrics residents and nurses, participated in the study. Initially a pretest questionnaire was given to each participant, followed by an educational intervention: a day’s worth of didactic lectures and practical training of practices for infection control. After two weeks, an identical post-test questionnaire was sent to the participants via email. Data were statistically analyzed through SPSS 22. *Z* test was applied to see the normality of data while paired t test was applied to compare the pretest score with posttest score.

**Results:**

Of 41 participants who attended the workshop, 34 participants responded to post-test giving an overall response rate of 83%. Out of 34, there were 27(80%) doctors and 7(20%) nurses, who participated in workshop. Each item of the questionnaire was analyzed, showing that pretest score for questions related to indication for hand washing, minimum timings required for hand rub, and spread of infection from unclean hands was quite low, as compared to post-test score, indicating statistically significant increment (p value 0.000, 0.001and 0.046 respectively). Mean pre-test score for doctors was 3.22 while for nurses, it was 3.14, whereas post-test score was 4.51 and 4.00 for doctors and nurses respectively. Overall, there was statistically significant increase in knowledge after educational intervention.

**Conclusion:**

There is statistically significant impact of educational intervention on improving the knowledge of Pediatric residents and nurses with respect to hand hygiene practices.

## INTRODUCTION

Healthcare associated infections (HAIs) are one of the leading nosocomial adverse events that not only cause of morbidity and mortality but also of monetary losses.^1^ A single most well-recognized, effective and simple way of decreasing HAIs is hand hygiene, yet healthcare professionals worldwide are not compliant with it.^2^ World Health Organization (WHO) estimates this compliance to be between 5% and 81%, with less than 40% average.^3^ This indicates the need for education of healthcare professionals by various means regarding hand hygiene. This education will instill and update the proper and much needed skill, knowledge and behavior regarding hand washing practices.^4^

The importance of educational intervention in augmenting and evolving knowledge and behavior is highlighted by many studies. Workshops for Pediatric infection prevention and control in hospital setting are periodically organized for Pediatric residents and nurses in the department of Pediatrics, King Edward Medical University/ Mayo hospital Lahore. The data in this study was collected after a one-day workshop focusing on the same subject.

## METHODS

As integral component of the periodic training program for the Pediatric residents and nurses, this questionnaire-based cross-sectional study was carried out in the department of Pediatrics, King Edward Medical University/ Mayo hospital Lahore in two weeks period. The study was approved by institutional review board.

The participants of workshop were 41 Pediatrics residents and nurses, belonged to 7 tertiary care institutions of Punjab. These participants had no previous formal training regarding infection control. A validated structured multiple answer based questionnaire was used. The construct of questions was to test the knowledge of the participants on hand hygiene. To prevent bias induced by participants’ apprehension, they were explained the purpose of the questionnaire and workshop and assured of full confidentiality. The consent of participants was obtained. A pretest questionnaire having 05 questions was handed out to each participant, followed by a full day program comprising two activities. Firstly, the senior teaching fraternity gave didactic presentations regarding control of nosocomial infection, specially emphasizing hand cleanliness. This was followed by second interventional activity which involved hands-on training about important routine practices for controlling infection including hand hygiene practices. Printed leaflets showing standard practices were circulated among participants. After two weeks, an identical post-test questionnaire was sent to the participants via email.

### Statistical analysis

Data on all the cases were subjected to statistical analysis through SPSS version 22 computer software. The responses were grouped as correct and incorrect responses. *Z* test was applied to see the normality of data. Every participant’s pre and post test scores were compared by applying *t* test to determine for improvement in knowledge about hand hygiene practices.

## RESULTS

Of 41 participants, attending the workshop, 34 participants responded to post-test giving an overall response rate of 83%. Out of 34, 27(80%) doctors participated in workshop while 7(20%) were nurses. Each item of the questionnaire was analyzed, showing that pretest score for questions related to indication for hand washing, minimum timings required for hand rub, and spread of infection from unclean hands was quite low, as compared to post-test score, indicating statistically significant increment (p-value 0.000, 0.001and 0.046 respectively) (Table-I). When separately analyzed for doctors and nurses, mean pre-test score for doctors was 3.22 while for nurses, it was 3.14, whereas post-test score was 4.51 and 4.00 for doctors and nurses respectively. It was observed that doctors responded well to this intervention, however, there was statistically significant increase in overall knowledge after educational intervention (Table-II).

**Table I:** Correct answers before & after educational intervention.

Question (Correct answer)	Doctors (27)			Nurses (07)				Total (34)		

	Pre-test	Post-test	p-value	Pre-test	Post-test	p-value	Pre-test	Post-test	p-value
***Most healthcare-associated pathogens are transmitted from patient to patient via (Hands of healthcare personnel)***	25 (92.6)	27 (100)	0.157	6 (85.7)	7 (100)	0.317	31 (91.2)	34 (100)	0.083
***WHO guidelines for hand hygiene recommend the use of hand washing with soap and water (When hands are visibly soiled)***	4 (14.8)	26 (95.3)	0.000	1 (14.3)	6 (85.7)	0.025	5 (14.7)	32 (94.1)	0.000
***What is the minimal time needed for alcohol-based handrub to kill most germs on your hands? (20 seconds)***	13 (48.1)	24 (88.9)	0.002	6 (85.7)	7 (100)	0.317	19 (55.9)	31 (91.2)	0.001
***Unclean hands don’t cause spread of which of the following organisms? (HIV)***	19 (81.5)	22 (85.3)	0.083	6 (85.7)	7 (100)	0.317	25 (73.5)	29 (85.3)	0.046
***Which of the following is the main route of cross-transmission of potentially harmful germs between patients in a health-care facility? (Health-care workers’ hands when not clean)***	23 (85.2)	26 (96.3)	0.083	2 (28.6)	2 (28.6)	1.000	25 (73.5)	28 (82.4)	0.083

**Table II:** Mean total score before & after educational intervention.

Profession		Mean	SD	SE	p-value
Doctor	Pre-Test	3.2222	0.75107	0.14454	0.000
	Post-Test	4.5185	0.64273	0.12369	
Nurses	Pre-Test	3.1429	0.37796	0.14286	0.001
	Post-Test	4.0000	0.00000	0.00000	

## DISCUSSION

Some hospitals organize short term programs for certified formal training in control of infection. This study was a part of a similar periodic program, focusing the training of Pediatric residents and nurses on hand hygiene practices. These participants had no previous formal training regarding infection control. So the pretest score revealed the baseline knowledge they had probably acquired during years of graduation or from their daily routine.

Expectedly, on average, the pretest scores were very low, showing poor prior knowledge about hand hygiene. However, the scores significantly improved (0.000 for doctors and 0.001 for nurses) after the instructive and hands-on sessions. This finding is consistent with the findings from Gaikwad UN et al.^1^ who found significant increase in the mean score after educational intervention. Fitzpatrick M et al.^5^ obtained similar results in their study which involved showing an instructional video to the participants on hand hygiene. Another one-day educational intervention study, focusing on disinfectant use by nurses, showed increment in the posttest scores.^6^ Nour-Eldein H et al.^7^ studied same in medical students and observed that educational intervention showed statistically significant increment in the post-test scores of students in all periods of the study. Our results are nearly similar to the outcome of a quasi-experimental research by Huang J et al.^8^ who reported that an educational intervention focusing on 100 randomly assigned nurses significantly enhanced their knowledge of universal precautions. The nurses received this intervention before and four months after their training. Comparable results were stated by Rezaee R et al.^9^ regarding medical students. However, contrary to these results, another intervention in remote past by Gould D et al.^10^ resulted in no beneficial effect of a hand hygiene training course, determined three months after the course. The contrast could be due to the difference in the tools used for assessment.

On analyzing individual items of the questionnaire, some items showed especially low pretest scores that showed notable increment after educational training. The results are similar from Gaikwad UN et al.^1^ This is unfortunate because it points towards a worrying deficiency in the residents’ and nurses’ knowledge of hygiene, and highlights the need for their repeated practical training concerning principals of general and hand hygiene.

Retention of knowledge after the educational activity is equally important as is the clinical implication of that knowledge. However, current study was not aimed to assess reduction in healthcare associated infections and compliance of residents and nurses for hand hygiene. This may be supposedly the most difficult part to evaluate since improvement in clinical practice may not be solely attributable to knowledge gain by one-day educational intervention. Moreover, a further follow-up survey after some time may help in deciding if the knowledge gain by the educational activity was helpful in retaining the knowledge for a longer period.

## CONCLUSION

The hands-on educational intervention has statistically significantly improved the knowledge of Pediatric residents and nursing staff with respect to hand hygiene practices. Educational interventions at periodic intervals should be encouraged to facilitate the knowledge on best practices, which may help to decrease the healthcare associated infections.

### Authors’ Contribution

**MFA and** **MHH** conceived, designed & editing of manuscript.

**MFA and AP** did data collection and manuscript writing.

**AH** did statistical analysis.

**MHH** did review and final approval of manuscript.
